# On the Relationship between the Indirectly Measured Attitude Towards Beer and Beer Consumption: The Role of Attitude Accessibility

**DOI:** 10.1371/journal.pone.0095302

**Published:** 2014-04-28

**Authors:** Mathilde Descheemaeker, Adriaan Spruyt, Dirk Hermans

**Affiliations:** 1 Faculty of Psychology and Educational Sciences, KU Leuven, Leuven, Belgium; 2 Faculty of Psychology and Educational Sciences, Ghent University, Ghent, Belgium; Erasmus University Rotterdam, Netherlands

## Abstract

Although some studies have demonstrated that the indirectly measured attitude towards alcohol is related to alcohol use, this relationship has not always been confirmed. In the current study, we attempted to shed light on this issue by investigating whether the predictive validity of an indirect attitude measure is dependent upon attitude accessibility. In a sample of 88 students, the picture-picture naming task, an adaptation of the affective priming paradigm, was used to measure the automatically activated attitude towards beer. Attitude accessibility was measured using a speeded evaluative categorization task. Behavioral measures were the amount of beer poured and drunk during a bogus taste test and the choice between a bottle of beer or water at the end of the experiment. In line with our hypothesis, the indirectly measured attitude towards beer predicted behavior during the taste test only when it was highly accessible. In contrast, this attitude was related to choice behavior irrespective of attitude accessibility. This study confirms that indirect attitude measures can be valuable predictors of alcohol-related behavior, but that it is sometimes necessary to take attitude accessibility into account.

## Introduction

Alcohol use can lead to a host of negative outcomes, which is most clearly seen in phenomena such as addiction and binge drinking. It is therefore important to study the psychological factors that are related to alcohol use. This knowledge will increase our understanding of the processes at play in alcohol abuse and addiction and will eventually lead to the development of prevention and treatment strategies.

One factor that has been related to alcohol use is the attitude one holds towards alcohol. Traditionally, attitudes are measured directly by means of self-report rating scales. However, the predictive validity of these direct attitude measures can be limited by a lack of introspective capacity [1] and measurement artifacts such as impression management and demand characteristics [2], [3]. This is not the case for more recently developed indirect attitude measures, which do not rely on self-report. Instead, an individual's automatically activated attitude towards certain (classes of) stimuli is inferred from the speed or accuracy with which the individual responds to these (classes of) stimuli during certain tasks. Well-known indirect attitude measures are the Implicit Association Test (IAT) [4], the Affective Priming Task (APT) [5], and the affect misattribution procedure [6]. As an example, consider the APT, which is used in the present study. This task requires participants to respond as quickly as possible to positive and negative target stimuli (e.g., to categorize these targets as positive or negative). Each target is preceded by a prime stimulus for which the attitudinal meaning is under investigation. There is reliable evidence showing that the evaluative connotation of the prime is automatically activated and that this leads to an affective priming effect: Performance is faster and more accurate when the prime and the target are affectively congruent than when these stimuli are affectively incongruent ([7], [8], see [9] for a meta-analysis). Accordingly, the APT can be used as a measure of participants' automatically activated attitudes towards the prime stimuli (e.g., [10]).

While these indirect attitude measures all aim to measure automatic attitude activation, it is clear that effects on these measures are driven by different underlying processes [11]–[13]. For example, some studies suggest that the APT captures the automatically activated attitude towards the specific exemplars used, while the IAT is more sensitive to the attitude towards categories of stimuli ([14], [15], but see [16], [17]).

Recent meta-analyses have demonstrated that indirect attitude measures are predictive of behavior over and above the predictive value of direct attitude measures [18], [19]. Also, Greenwald et al. [19] showed that the degree of social sensitivity of a research topic limits the predictive validity of a direct attitude measure to a much greater extent than it does that of an indirect attitude measure. As alcohol use can be a socially sensitive topic and automatic processes have been shown to play an important role in problematic drinking [20], [21], indirect attitude measures are increasingly used in this research domain. There is now substantial evidence showing that alcohol use is related to the indirectly measured attitude towards alcohol (mostly measured with the IAT), even after controlling for the variance explained by the self-reported attitude [19], [22]–[24]. Thush and Wiers [25], for example, observed that an IAT measure of the attitude towards alcohol was predictive of the frequency of binge drinking one year later in a sample of adolescents. Moreover, this effect was found over and above the variance explained by a direct measure of alcohol outcome expectancies. Similarly, Payne, Govorun, and Arbuckle ([26], Study 1) showed that the attitude towards beer relative to water as measured by the affect misattribution procedure predicted the choice students made between beer and water.

It may be noted, however, that most studies conducted so far have relied on self-report measures of alcohol use as a dependent variable. Clearly, this type of measure is subject to the same biases and limitations as direct attitude measures [27]. Moreover, the relationship between indirect attitude measures and (often self-reported) alcohol use has not always been confirmed [23], [24]. For example, in a recent set of three studies, an IAT measure of the attitude towards alcohol failed to predict alcohol consumption observed in a semi-naturalistic setting, although it was related to self-reported binge drinking and weekly consumption in two of these studies [28].

The mixed evidence concerning the contribution of indirect attitude measures to the prediction of alcohol use has initiated research into the moderators of this relationship. For example, several studies have provided evidence that indirect attitude measures are more predictive of alcohol-related behavior when the capacity for self-control is low [29]–[31]. Surprisingly little attention has been paid, however, to the extensive literature in the domain of social cognition concerning the determinants of attitude-behavior consistency. In this tradition, attitude characteristics such as attitude stability, ambivalence, and accessibility have repeatedly been shown to moderate the relationship between directly measured attitudes and behavior (see [32]–[34] for meta-analyses). Nevertheless, this literature is seldom drawn upon in the field of experimental psychopathology in general and when using indirectly measured attitudes as a predictor of alcohol use in particular.

Therefore, the aim of the present study was to examine whether attitude accessibility acts as a moderator of the relationship between the indirectly measured attitude towards alcohol and alcohol use. Attitude accessibility is a component of attitude strength and refers to the ease with which an attitude can be retrieved from memory [35]. Fazio and colleagues (e.g., [5]) proposed an attitude-nonattitude continuum ranging from attitudes that have to be constructed on the spot (because there is no a priori evaluation available) to highly accessible attitudes. Crucially, while there is ample evidence showing that the consistency between self-reported attitudes and behavior is higher when these attitudes are more accessible ([36], see [32]–[34] for meta-analyses on this topic), it remains largely unexplored whether this is also the case when attitudes are measured indirectly. The only study to our knowledge that relates to this research question showed that a self-activation manipulation (which is assumed to increase the accessibility of self-related knowledge) increased the predictive validity of the IAT for several behaviors, including self-reported alcohol consumption [37].

### Current Study

Because alcohol is a heterogeneous category and the attitudes towards subcategories can differ, we decided to focus on beer in the current study. Not only did we investigate the relationship between the indirectly measured attitude towards beer and self-reported beer consumption, we also attempted to predict behavior that was registered during the experiment. More specifically, we registered (a) the amount of beer relative to water participants poured and (b) drank during a bogus taste test and (c) whether participants chose to take home a bottle of beer or water at the end of the experiment. Behaviors during the taste test were relative (beer in comparison to water) to control for factors that can influence beer consumption but are not related to the attitude towards beer (e.g., thirst).

Attitude accessibility is typically measured as the response latency of evaluation: The faster an attitude object can be evaluated, the more accessible the attitude towards this object [36]. In line with previous studies (e.g., [38]–[40]), we controlled for inter-individual differences in the speed of evaluation that are unrelated to differences in attitude accessibility. This was done by operationalizing the accessibility of the attitude towards beer as the difference in response latency between the evaluative categorization of beer stimuli (positive versus negative) and the non-evaluative categorization of beer stimuli (beer versus water, hereafter referred to as semantic categorization).

As an indirect measure of the attitude towards beer relative to water, we opted for the picture-picture naming task (PPNT) [41]. In this version of the APT, primes and targets are pictures and participants are asked to name the targets as quickly as possible. This task has been shown to predict behavior over and above direct attitude measures in previous research [41]–[43]. We hypothesized that the predictive validity of the PPNT would be most pronounced in participants whose attitude towards beer was highly accessible.

## Method

### Ethics Statement

This study was approved by the ethical committee of the KU Leuven and all participants gave written informed consent.

### Participants

Eighty-eight first-year psychology students participated in partial fulfillment of a course requirement (70 women; age: *M* = 18.6, *SD* = 0.8). To avoid self-selection bias, students were not informed that they would be asked to taste beer before they signed up. All participants were native Dutch speakers and had normal or corrected-to-normal vision. Because a small amount of alcohol was to be consumed during the experiment, we used the following exclusion criteria: pregnancy, past or current alcohol abuse, use of medication that should not be combined with alcohol, and a medical condition that contraindicates alcohol consumption.

### Apparatus and Stimuli

Computer tasks were run on an AMD Athlon XP computer with a 16-inch CRT monitor (85 Hz, resolution 1024×768). Affect 4.0 software [44] controlled stimulus presentation and registered responses. Additionally, we used a response box with two keys during the categorization tasks and an external voice key during the PPNT.

All visual stimuli used during the experiment had a dimension of 512 by 384 pixels and were presented against the black background of the computer monitor. As PPNT targets, we selected four positive pictures (bride, Christmas tree, puppy, sun) and four negative pictures (fire, gun, trash, worms) on the basis of a preliminary study during which 51 students rated the affective connotation of 215 real life color pictures [45]. Each target picture could be named with a single Dutch word. Participants in the current experiment rated the positive targets as significantly more positive than the negative targets on a scale ranging from −10 (*very negative*) to 10 (*very positive*), *M*_positive_ = 7.66 (*SD* = 0.65), *M*_negative_ = −7.84 (*SD* = 0.63), *t*(6)  = 34.12, *p*<.001. Primes were eight pictures of beer (seven of which clearly depicted a brand) and eight pictures of still water (seven of which clearly depicted a brand). These beverages were presented on a white background in order to reduce the influence of stimulus characteristics unrelated to the type of beverage. For the practice trials, we used four neutral pictures (cup, glasses, hammer, spoon) as primes. During the attitude accessibility measure (evaluative and semantic categorization tasks), participants were presented with the same eight beer and eight water pictures used during the PPNT. Additionally, during the evaluative categorization task, we used eight clearly positive pictures and eight clearly negative pictures as filler stimuli. We used different stimuli during the practice trials (two positive and two negative pictures for the evaluative categorization task and two beer and two water pictures for the semantic categorization task). Positive, negative, and neutral pictures were also selected on the basis of the previously mentioned study by Spruyt et al. [45].

### Procedure

Participants were tested individually in a sound-attenuated room and the lights were dimmed during the computer tasks. The experiment was introduced as an investigation of the evaluation of different brands of beer and still water. The experiment proceeded as follows: Participants first completed the attitude accessibility measure, followed by a filler task, the PPNT, a direct attitude measure, the bogus taste test, a questionnaire concerning beer and alcohol consumption, and the choice of a beverage. To reduce the influence of the time of day on beer consumption during the taste test, the experiment always took place in the afternoon or evening.

To measure *attitude accessibility*, we asked participants to complete two speeded categorization tasks. The first was an evaluative categorization task, during which participants were instructed to categorize stimuli as positive or negative as quickly as possible using the response box. After this, participants completed a semantic categorization task, during which stimuli had to be categorized as beer or water. As described above, these stimuli were eight beer and eight water pictures. During the evaluative categorization task, eight positive and eight negative pictures were added as filler stimuli. Both tasks were made up of two blocks and each stimulus was presented once per block in a semi-random order (no more than two consecutive beer or water stimuli and no more than three consecutive filler stimuli). This resulted in 64 evaluative categorization trials and 32 semantic categorization trials. These trials started with a 500 ms presentation of a fixation cross and a 500 ms interval, followed by the presentation of the stimulus until participants responded. The inter-trial interval varied between 500 and 1500 ms with an average of 1000 ms. The assignment of the left and the right key of the response box to the responses positive/negative and beer/water was counterbalanced across participants. Before the experimental trials, participants received eight practice trials for each task (the practice stimuli described above were all presented twice). The experimenter was not present during the experimental trials.

Because participants expressed their attitude towards beer and water repeatedly during the evaluative categorization task and attitude rehearsal has been shown to strengthen attitude accessibility [36], one might argue that this task was not only a measure, but also an (unintended) manipulation of attitude accessibility. To reduce this influence, we administered a *filler task* (a simple experiment on causal learning of the type reviewed by De Houwer and Beckers [46]) before continuing the experiment. This task lasted approximately 15 minutes.

We used the *PPNT* as an indirect attitude measure. This task started with three series of practice trials. First, participants viewed all the targets (four positive and four negative pictures) one by one, together with their correct name. They were instructed to read the name of each target out loud, to remember it, and to press the space bar when they were ready for the next target. During the second series of practice trials, the targets were presented a second time, but without their corresponding names. Participants were asked to name each target as quickly as possible. During these and all subsequent trials, a trial ended when the voice key was triggered or 2000 ms had elapsed. The experimenter used the keyboard to code whether the voice key had been triggered accurately and whether the participant's response was correct. The third series of practice trials was identical to the actual priming trials, but four neutral pictures were used as primes and each target was presented twice. During the actual priming phase, all primes (eight beer and eight water pictures) and targets were combined, resulting in 128 trials. Participants were instructed to continue naming the targets as quickly as possible, without taking the primes into account. Each trial started with a 500 ms presentation of a fixation cross and a 500 ms interval, after which the prime was presented for 200 ms. Immediately after the prime, the target was presented, resulting in a stimulus onset asynchrony of 200 ms. The inter-trial interval varied between 500 and 1500 ms with an average of 1000 ms. The actual priming trials were divided into four blocks (32 trials per block) in such a way that each block contained the same amount of the different trial types. Within these blocks, trials were presented in a random order. Blocks were separated by instructions reminding participants to name the targets as quickly as possible.

As a final computer task, participants filled out several rating scales. They were asked to evaluate the target and the prime pictures on a scale ranging from −10 (*very negative*) to 10 (*very positive*). On the same scale, they indicated their evaluation of beer, alcohol, and water in general. They also reported how much they liked drinking beer, alcohol, and water on a scale ranging from 0 (*not at all*) to 10 (*very much*). With the exception of the evaluation of the targets, these rating scales were used to *measure attitudes directly*.

Next, during the *bogus taste test*, participants were asked to fill out a form concerning their ratings of three unidentified brands of beer and three unidentified brands of still water on different taste-related characteristics. The order of beer and water on the form was counterbalanced across participants. We used beer low in alcohol content (alcohol by volume<1%), but participants were not informed of this. At the end of the experiment, participants were asked to estimate the alcohol content of the tasted beers on a scale ranging from 0 (*no alcohol*) to 6 (*very high alcohol content*). Only one participant gave a score of 0, six participants gave a score of 1, and the average rating was 2.87 (*SD* = 1.03). We presented participants with three pitchers of beer and three pitchers of water, each filled with 125 (±2) grams of liquid, and a plastic cup for each beverage. Unbeknown to participants, behavioral measures were the amount of beer drunk and poured subtracted by the amount of water drunk and poured respectively. We told participants that we would throw away what was left in the pitchers and that they could drink more than was necessary to fill out the taste test form. The experimenter was not present during this task and participants could take as much time as they wanted.

At the end of the experiment, participants filled out a *questionnaire about beer and alcohol consumption*. This questionnaire consisted of eight items relating to the frequency, amount, and negative consequences of the consumption of beer and alcohol (assessed separately) in daily life.

As a final behavioral measure, participants were presented with the *choice between a bottle of beer* (with a standard alcohol content) *and a bottle of water* to take home. They were told this was a small token of appreciation for their participation. Participants made this choice when leaving the lab and the experimenter was in an adjacent room at this time.

## Results

### Data Reduction

Four participants were excluded from the analyses: one participant because of an unusually large amount of errors (50%) during the semantic categorization task, one participant because of an experimenter error during the administration of the taste test, and two participants because we were not able to accurately register a response on more than 25% of the PPNT trials (due to technical errors in the activation of the voice key, naming errors, or the absence of a response within the 2000 ms response window). This resulted in a sample of 84 students (68 women) between 18 and 23 years old (*M* = 18.6, *SD* = 0.8). All data can be obtained by contacting the corresponding author.

With regard to the categorization tasks, two trials with a response latency of 1 ms and two trials during which a participant interrupted the task to ask a question were not included in the analyses. In addition, semantic categorization trials on which an incorrect response was given (categorizing beer as water or vice versa; 1.7%) were excluded. Finally, to reduce the impact of outlying values, we discarded response latencies that deviated more than 2.5 standard deviations from a participant's mean latency for each trial type (e.g., evaluative categorization trials with beer stimuli; 2.6%; see [47]). The accessibility of the attitude towards beer was calculated by subtracting the mean reaction time of semantic categorization of beer stimuli from the mean reaction time of evaluative categorization of beer stimuli (this means that a higher score corresponds with a less accessible attitude). The mean accessibility score was 408 ms with a standard deviation of 300 ms. We performed a tertile split to create a high, a moderate, and a low accessibility group.

We also excluded certain PPNT trials when calculating an affective priming score. If a participant rated a positive target as negative or vice versa, all trials with this target were excluded for this participant (this occurred six times). Trials on which a target was incorrectly named (1.7%), the voice key was not appropriately activated (5.0%), or no response had been given when 2000 ms had elapsed (0.1%) were also excluded. As for the categorization tasks, we discarded response latencies that deviated more than 2.5 standard deviations from a participant's mean latency for each trial type (e.g., trials with a beer prime followed by a positive target; 1.8%; see also [41]). Based on mean reaction times (RT) per trial type, the affective priming score was calculated as follows: (mean RT beer/negative – mean RT beer/positive) – (mean RT water/negative – mean RT water/positive). In this way, higher priming scores reflect a more positive automatically activated attitude towards beer relative to water. The mean priming score was −0.70 ms with a standard deviation of 25.63 ms. The internal consistency of this measure was determined by calculating the priming score for each beer prime relative to water (e.g., Stella: [mean RT Stella/negative – mean RT Stella/positive] – [mean RT water/negative – mean RT water/positive]) and subsequently computing Cronbach's α of a scale consisting of these priming scores. Internal consistency was acceptable for a performance-based measure (Cronbach's α of .63).

A composite score reflecting the directly measured attitude towards beer was calculated by averaging the evaluation of beer, the liking rating of beer (after transformation to a scale ranging from −10 to 10), and the mean evaluation of the beer primes. The directly measured attitude towards water was calculated in the same way. In line with the affective priming score, we used a relative measure that was calculated by subtracting the attitude towards water from the attitude towards beer. The mean of this direct measure of the attitude towards beer relative to water was −4.29 and the standard deviation was 6.02.

Due to the high correlation between the amount of beer relative to water poured (*M* = −51.83 g, *SD* = 56.61 g) and drunk (*M* = −54.27 g, *SD* = 52.74 g) during the taste test, *r*(82) = .92, *p*<.001, we created a composite measure by summing the standardized scores. To obtain a measure of self-reported beer consumption, we calculated the sum score of the standardized beer-related items of the questionnaire about beer and alcohol consumption. This measure demonstrated high internal consistency (Cronbach's α of .92).

### Behavioral Prediction

To investigate whether the affective priming score explained unique variance in behavior, we conducted hierarchical regression analyses in which we controlled for the effects of gender and the directly measured attitude, which are known predictors of alcohol use. In this regard, it is relevant to mention that the directly and indirectly measured attitude towards beer relative to water were not correlated, *r*(82) = −.00, *p* = .966. We examined the moderating role of the accessibility of the attitude towards beer by testing whether the interaction effect between the accessibility and the priming score was significant. To that end, predictors were centered at their means in order to avoid multicollinearity. All significance tests were two-tailed.

#### Behavior during the taste test

Linear regression analysis (see Table 1) indicated that the affective priming score did not predict behavior during the taste test in the group as a whole. Since the interaction effect between the accessibility score and the priming score only just missed the conventional level of significance, we examined the predictive validity of the PPNT in each of the three accessibility groups (see Table 2). In line with our hypothesis, the priming score was a significant predictor of behavior during the taste test in the high accessibility group, but not in the moderate or low accessibility group. The standardized DFBeta statistic revealed one influential case (absolute value larger than 1) in the low accessibility group, but the effect of the priming score remained nonsignificant after excluding this participant.

**Table 1:** Hierarchical multiple linear regression analyses for the prediction of behavior during the taste test.

Predictor	Δ*R* ^2^	*P*	β	*t*	*p*
Step 1	.10	.017			
Gender^a^			0.02	0.20	.845
Direct attitude measure: beer relative to water^b^			0.32	2.79	.006
Step 2	.01	.381			
PPNT: beer relative to water			0.09	0.88	.381
Step 3	.00	.715			
Accessibility of the attitude towards beer^c^			−0.04	−0.37	.715
Step 4	.04	.060			
PPNT x Accessibility			−0.26	−1.91	.060

*Note.* Behavior during the taste test was the sum of the standardized amount of beer relative to water poured and the standardized amount of beer relative to water drunk. Predictors were centered at their means. PPNT = picture-picture naming task.

a Coded as 0 = male and 1 = female.

b Calculated by averaging the evaluation rating, liking rating (after transformation to a scale ranging from −10 to 10), and mean evaluation rating of the primes for beer and water separately and subsequently subtracting the composite score for water from the composite score for beer.

c Calculated by subtracting the mean reaction time of categorization of beer stimuli as beer from the mean reaction time of evaluative categorization of beer stimuli.

**Table 2:** Hierarchical multiple linear regression analyses for the prediction of behavior during the taste test in the three accessibility groups.

Predictor	Δ*R* ^2^	*P*	β	*t*	*p*
High accessibility group (*n* = 28)					
Step 1	.04	.621			
Gender^a^			−0.15	−0.72	.476
Direct attitude measure: beer relative to water^b^			0.08	0.38	.705
Step 2	.25	.008			
PPNT: beer relative to water			0.50	2.89	.008
Moderate accessibility group (*n* = 28)					
Step 1	.09	.291			
Gender^a^			0.04	0.19	.855
Direct attitude measure: beer relative to water^b^			0.32	1.58	.128
Step 2	.00	.742			
PPNT: beer relative to water			−0.07	−0.33	.742
Low accessibility group (*n* = 28)					
Step 1	.20	.065			
Gender^a^			0.19	0.96	.346
Direct attitude measure: beer relative to water^b^			0.48	2.47	.021
Step 2	.02	.413			
PPNT: beer relative to water			−0.15	−0.83	.413

*Note.* Behavior during the taste test was the sum of the standardized amount of beer relative to water poured and the standardized amount of beer relative to water drunk. Groups were created by means of a tertile split. PPNT = picture-picture naming task.

a Coded as 0 = male and 1 = female.

b Calculated by averaging the evaluation rating, liking rating (after transformation to a scale ranging from −10 to 10), and mean evaluation rating of the primes for beer and water separately and subsequently subtracting the composite score for water from the composite score for beer.

#### Choice behavior

Fifty-eight participants chose to take home a bottle of water, 21 participants opted for a bottle of beer, and five participants did not make a choice. Logistic regression analysis (see Table 3) showed that the affective priming score was significantly related to participants' choice. There was no evidence that the accessibility of the attitude towards beer moderated this relationship, as the interaction effect between the accessibility and the priming score was not significant (and results were similar in the three accessibility groups).

**Table 3:** Hierarchical multiple logistic regression analyses for the prediction of choice behavior.

Predictor	Δ*R* ^2^ a	χ^2^	*p*	*OR* b	*Wald*	*p*
Step 1	.26	15.82	<.001			
Gender^c^				0.74	0.19	.664
Direct attitude measure: beer relative to water^d^				3.43	8.27	.004
Step 2	.10	6.65	.010			
PPNT: beer relative to water				2.21	5.76	.016
Step 3	.00	0.10	.755			
Accessibility of the attitude towards beer^e^				0.90	0.09	.759
Step 4	.01	0.45	.502			
PPNT x Accessibility				1.35	0.46	.497

*Note.* Choice behavior was coded as 0 = water and 1 = beer. Continuous predictors were standardized in order to facilitate the interpretation of the odds ratio. PPNT = picture-picture naming task.

a Nagelkerke *R* ^2^.

b The odds ratio of, for example, the PPNT score can be interpreted as follows: When the PPNT score increases by one standard deviation, the odds of choosing beer increase by a factor of 2.21 (given that gender and the directly measured attitude are held at a fixed value).

c Coded as 0 = male and 1 = female.

d Calculated by averaging the evaluation rating, liking rating (after transformation to a scale ranging from −10 to 10), and mean evaluation rating of the primes for beer and water separately and subsequently subtracting the composite score for water from the composite score for beer.

e Calculated by subtracting the mean reaction time of categorization of beer stimuli as beer from the mean reaction time of evaluative categorization of beer stimuli.

#### Self-reported beer consumption

The affective priming score was not related to beer consumption in daily life as measured by the previously described questionnaire (see Table 4). There was also no evidence for a moderating role of attitude accessibility, as the interaction effect between the accessibility and the priming score was not significant (and results were similar in the three accessibility groups). Conclusions remained the same when we did not control for gender and the direct attitude measure and thus used only the affective priming score as a predictor.

**Table 4:** Hierarchical multiple linear regression analyses for the prediction of self-reported beer consumption.

Predictor	Δ*R* ^2^	*p*	β	*t*	*p*
Step 1	.48	<.001			
Gender^a^			−0.04	−0.48	.632
Direct attitude measure: beer relative to water^b^			0.68	7.86	<.001
Step 2	.00	.770			
PPNT: beer relative to water			−0.02	−0.29	.770
Step 3	.02	.071			
Accessibility of the attitude towards beer^c^			−0.15	−1.83	.071
Step 4	.00	.564			
PPNT x Accessibility			0.06	0.58	.564

*Note.* Self-reported beer consumption was measured with a questionnaire that assessed amount, frequency, and negative consequences of beer consumption. Predictors were centered at their means. PPNT = picture-picture naming task.

a Coded as 0 = male and 1 = female.

b Calculated by averaging the evaluation rating, liking rating (after transformation to a scale ranging from −10 to 10), and mean evaluation rating of the primes for beer and water separately and subsequently subtracting the composite score for water from the composite score for beer.

c Calculated by subtracting the mean reaction time of categorization of beer stimuli as beer from the mean reaction time of evaluative categorization of beer stimuli.

#### Direct attitude measure

Although the predictive validity of the direct attitude measure was not the focus of the present research, it is worth pointing out that, in contrast to what we would have expected, the direct attitude measure was predictive of behavior during the taste test only in the low accessibility group (see Table 2). In line with this finding, the interaction effect between attitude accessibility and the direct attitude measure was significant, β = 0.29, *t*(79)  = 2.43, *p* = .017. To ensure that differences found between the three accessibility groups in the relationship of the PPNT with behavior during the taste test were not due to differences in the amount of variance already explained by the direct attitude measure, we repeated these analyses without controlling for the direct measure. Results were virtually identical: The affective priming score was predictive only in the high accessibility group.

Because of these unexpected results, we also tested this interaction effect for the other dependent variables. The relationship between choice behavior and the direct attitude measure was moderated by attitude accessibility in the expected direction, *OR* = 0.27, *Wald* = 6.09, *p* = .014. The standardized DFBeta statistic for the interaction effect revealed one influential case (absolute value larger than 1), but the interaction effect remained significant after excluding this participant. There was no evidence for a moderating role of attitude accessibility when predicting self-reported beer consumption, |*t|*<1.

## Discussion

Although some studies have shown the indirectly measured attitude towards alcohol to be related to alcohol use (e.g., [25], [26]), other studies failed to provide corroborating evidence for this relationship (e.g., [28]; see [23] for a review). Drawing upon the social cognition literature concerning the moderators of the consistency between self-reported attitudes and behavior, we attempted to clarify these inconsistent results by investigating whether the predictive validity of an indirect attitude measure is dependent upon attitude accessibility. Results obtained when predicting the amount of beer poured and drunk during a bogus taste test confirmed our hypothesis: The attitude towards beer as measured by the PPNT was predictive in participants whose attitude towards beer was highly accessible, but not in participants who exhibited moderate or low levels of attitude accessibility. To our knowledge, this study is the first to demonstrate that attitude accessibility moderates attitude-behavior consistency when attitudes are measured indirectly. This finding offers a possible explanation as to why previous studies failed to find a relationship between indirectly measured attitudes and observed alcohol consumption, as these studies did not take attitude accessibility into account [28].

We did not find evidence, however, for a similar moderation effect when predicting the choice between a bottle of beer and a bottle of water at the end of the experiment. That is, the indirectly measured attitude towards beer was related to choice behavior irrespective of attitude accessibility. To account for this set of observations, one might argue that attitude accessibility will have less impact on attitude-behavior consistency when there is sufficient motivation and opportunity to engage in deliberative processing [36]. Unlike pouring and drinking behavior, choice behavior requires an explicit consultation of one's attitudes towards the different choice alternatives. As a result, a reduced impact of attitude accessibility can be expected. Alternatively, it could be argued that the absence of a moderation effect when looking at the behavioral choice data simply reflects a type II error. The observation that attitude accessibility did moderate the relationship between choice behavior and the *direct* attitude measure is consistent with this viewpoint. Irrespectively, it would be interesting to investigate whether the degree to which attitude accessibility impacts the relationship between an indirect attitude measure and behavior is itself moderated by the extent to which the criterion behavior requires deliberative processing.

An important advantage of the current study is that we did not rely solely on self-reported alcohol consumption, as was the case in the majority of previous studies on the relationship between indirect attitude measures and alcohol use, but also included measures of alcohol-related behavior registered during the experiment. The present study suggests that this is an important issue. Whereas the PPNT was able to predict behavior registered during the experiment, PPNT scores were unrelated to self-reported beer consumption. This data pattern is in line with the findings of Spruyt et al. [41], who used the PPNT as an indirect measure of the attitudes towards fruit and candy bars. In their study, the PPNT was found to be predictive of the choice between an apple or a Snickers candy bar at the end of the experiment, but was not related to self-reported consumption of these products. Interestingly, Spruyt et al. [41] also administered a candy/fruit IAT and found the exact opposite data pattern with this measure: The IAT was clearly related to self-reported consumption, but not to the actual choice behavior monitored during the experiment. Previous studies that did find evidence for a relationship between the indirectly measured attitude towards alcohol and self-reported drinking and/or drinking problems also used the IAT or the Extrinsic Affective Simon Test as an indirect attitude measure [23]. Taken together, it can be hypothesized that finding a relationship between an indirect attitude measure and self-reported alcohol consumption is dependent on the specific measure used. Evidence that different indirect attitude measures rely on different underlying processes (e.g., [11], [12]) is clearly consistent with this reasoning. It would thus be interesting to examine whether different indirect attitude measures are predictive of different outcome measures.

Although not the focus of the current study, it may be noted that the accessibility of the attitude towards beer also moderated the relationship of the direct attitude measure with behavior during the taste test and choice behavior. To our surprise, however, the directly measured attitude towards beer was predictive of behavior during the taste test only in individuals who exhibited a low level of accessibility. This finding contrasts with previous studies showing a stronger relationship between direct attitude measures and behavior when attitudes are more accessible [32]–[34]. Moreover, when predicting choice behavior, we found a moderating effect in the expected direction. Therefore, it is probably wise not to put too much weight on this isolated finding.

A limitation of the current study is that we used a specific sample of largely female, first-year university students. Even though the processes under study are assumed to be universal, we cannot be sure that our findings generalize to the broader population. In particular, more research is needed to verify whether our findings generalize to a clinical population suffering from alcohol abuse or addiction.

In summary, we demonstrated that attitude accessibility can moderate the predictive validity of an indirect attitude measure. In addition, our findings suggest that the degree to which this moderating effect occurs might itself be dependent upon the precise nature of the behavior being examined. Although further research is needed to substantiate the latter claim, the results of the current study clearly indicate that future research concerning indirectly measured attitudes towards alcohol as a predictor of alcohol use should focus not only on the evaluative quality of these attitudes, but also on their accessibility.
